# Oral Pigmentation in Children: A Narrative Review and Practical Diagnostic Framework

**DOI:** 10.7759/cureus.115604

**Published:** 2026-09-01

**Authors:** Otgontsetseg Dagvadorj, Damdindorj Boldbaatar, Nursabi Khizatkhan, Altansukh Tsend-Ayush, Uurtuya Shuumarjav, Dariimaa Ganbat, Delgertsetseg Jargaltsogt

**Affiliations:** 1 Communication Skills, Mongolian National University of Medical Sciences, Ulaanbaatar, MNG; 2 Administration, Mongolian National University of Medical Sciences, Ulaanbaatar, MNG; 3 Medical Sciences, Mongolian National University of Medical Sciences, Ulaanbaatar, MNG; 4 Dental Hygiene, Mongolian National University of Medical Sciences, Ulaanbaatar, MNG

**Keywords:** gingival pigmentation, oral melanotic macule, oral mucosal melanoma, oral pigmentation, pediatric dentistry, peutz-jeghers syndrome, primary adrenal insufficiency

## Abstract

Oral pigmentation in childhood ranges from common constitutional variants to less frequent reactive, local, medication-related, vascular, genetic, endocrine, and neoplastic disorders. Overlapping clinical appearances can create diagnostic uncertainty, especially when adult-oriented approaches are applied to children. This narrative review presents a pediatric-focused framework for initial evaluation based on five domains: onset and evolution; focal, multifocal, or diffuse distribution; color and surface morphology; blanching or compressibility; and medication, dental, family, environmental, and systemic clues. Longstanding, symmetric, diffuse brown pigmentation without symptoms usually favors constitutional melanin pigmentation. Multifocal lentigines with gastrointestinal, endocrine, skeletal, or family-history findings should prompt targeted evaluation for an underlying syndrome. Blue-gray lesions adjacent to a restoration or trauma site suggest exogenous pigment, whereas red-blue compressible lesions raise concern for a vascular process and should not undergo blind biopsy. Oral melanoacanthoma is an important benign mimic because it may enlarge rapidly and appear clinically alarming. New or changing focal lesions, particularly those showing nodularity, ulceration, spontaneous bleeding, marked asymmetry, irregular borders, color variegation, or diagnostic uncertainty, warrant prompt specialist assessment and histopathologic evaluation when indicated. Pediatric evidence remains limited and consists largely of observational studies, case reports or series, and extrapolation from adult literature; accordingly, the proposed pathway is an evidence-informed clinical aid rather than a validated prediction rule. A structured history-first approach can support appropriate reassurance, targeted referral, and timely biopsy while minimizing unnecessary intervention for convincingly benign pigmentation.

## Introduction and background

Oral mucosal pigmentation is a visible clinical finding rather than a single disease entity. Pigment may arise from melanin, blood or its breakdown products, exogenous material, medications, or less common metabolic and neoplastic processes. Contemporary reviews emphasize that the clinical spectrum extends from harmless physiologic pigmentation to disorders that require systemic investigation or biopsy [1-4]. In children, the diagnostic problem is modified by age, developmental stage, the relative rarity of malignancy, the importance of congenital and genetic disorders, and the need to avoid unnecessary invasive procedures.

Pediatric-specific evidence is comparatively sparse. A report in Archives of Disease in Childhood illustrates the diagnostic difficulty of childhood oral pigmentation, while observational work demonstrates variation in gingival melanin expression across children with different skin tones and clinical backgrounds [5,6]. Parental smoking has also been associated with greater gingival melanin pigmentation in a pediatric cross-sectional study, but such findings are hypothesis-generating and should not be used to diagnose secondhand-smoke exposure [7].

A pediatric approach should therefore place greater weight on the timing and evolution of pigmentation and on the clinical context. Longstanding, stable constitutional pigmentation can often be documented and observed, whereas genuinely new diffuse pigmentation, a new focal lesion, or pigmentation accompanied by systemic clues deserves a lower threshold for further evaluation. Several acquired explanations common in adults, including smoker's melanosis and long-standing restorative-material tattoos, are less typical in younger children [1,2].

This narrative review aims to convert the broad differential diagnosis into a practical pediatric clinical pathway. The emphasis is not on memorizing every cause of oral discoloration, but on recognizing patterns that separate lesions suitable for documentation and observation from those requiring targeted systemic evaluation, oral medicine referral, imaging, or biopsy. The proposed framework is intended to support structured clinical decision-making rather than replace specialist assessment or histopathology when diagnostic uncertainty persists.

## Review

Scope and approach of this narrative review

This article is a clinically oriented narrative synthesis rather than a systematic review. Pediatric observational studies, case reports and case series, systematic reviews, and pediatric management guidance were prioritized when available; adult or mixed-age oral-medicine literature was used when pediatric data were sparse. Sources were selected for their relevance to pediatric presentation, differential diagnosis, first-line management, and clinical safety. No protocol registration, formal risk-of-bias assessment, or quantitative pooling was undertaken. The framework should therefore be interpreted as evidence-informed decision support rather than a validated diagnostic algorithm.

Clinically useful classification

A practical first step is to classify pigmentation along three axes: source, distribution, and clinical risk. By source, pigmentation may be endogenous (most commonly melanin or blood-related pigment) or exogenous (for example, dental material or traumatic implantation). By distribution, it may be focal, multifocal, or diffuse. By clinical risk, it can be provisionally grouped into a typical benign pattern, a pattern suggesting systemic disease, an exogenous or vascular mimic, or an indeterminate or suspicious lesion requiring tissue diagnosis [2-4,8].

Before applying this classification, the clinician should confirm that the discoloration is mucosal rather than a tooth-surface stain. In children, dark extrinsic dental staining may be mistaken for mucosal pigmentation; careful inspection and the response to professional cleaning can usually resolve this distinction. Color alone is not a diagnostic tool. Superficial melanin commonly appears brown to black, deeper pigment can appear blue-gray, vascular lesions may appear red, purple, or blue, and exogenous dental material may produce a blue-gray macule. The combination of color, location, symmetry, surface, duration, and clinical context is more informative than any single visual feature [1-4].

Constitutional and reactive melanin pigmentation

Constitutional (physiologic) melanin pigmentation is the most common benign explanation for diffuse oral pigmentation. It generally appears as bilateral, symmetric, flat brown-to-dark-brown pigmentation, often involving the attached gingiva and other oral sites. Melanin pigmentation reflects melanocyte activity rather than a simple increase in melanocyte number, and its intensity varies substantially among individuals [3,4,8,9]. In a pediatric observational study of 100 children, skin color was useful in predicting pigmentation intensity, although distribution and intensity were not consistently associated with age or sex [6].

When the pattern is longstanding, symmetric, asymptomatic, and consistent with the child's constitutional pigmentation, reassurance and baseline photographic documentation are usually appropriate. Atypical features - particularly recent onset, rapid change, asymmetry, a discrete solitary lesion, nodularity, ulceration, or bleeding - should shift the clinician away from a purely physiologic explanation [1-4].

Reactive pigmentation should also be considered. Post-inflammatory melanosis may follow mucosal inflammation, ulceration, or trauma and typically occupies the site of the preceding lesion [1,2]. In adolescents, direct tobacco use should be included in the history because smoker's melanosis is a recognized acquired pattern in literature about oral medicine. Environmental tobacco-smoke exposure warrants separate caution: a 2024 pediatric study found an association between parental smoking and gingival melanin pigmentation [7], but the cross-sectional, questionnaire-based design does not support using pigmentation as a stand-alone exposure biomarker. When precise exposure classification is required for research, objective biomarkers such as cotinine are preferable.

Solitary melanotic lesions: melanotic macule, melanocytic nevus, and melanoacanthoma

Solitary brown or black lesions deserve a lesion-specific assessment. The melanotic macule is a common benign pigmented lesion in oral pathology series, whereas oral melanocytic nevi are substantially less frequent [10,11]. In a pediatric case series of 10 Latin American children with solitary pigmented oral lesions, the gingiva and palate were the most common sites; lesions were flat and smaller than 1 cm, and histopathology was required to distinguish melanotic macules from nevi and exogenous pigmentation [10].

Large clinicopathologic data from mixed-age populations also demonstrate the limitations of visual diagnosis. In a series of 773 solitary melanocytic lesions, melanotic macules represented the great majority, whereas nevi and melanoma were uncommon; the palate was a frequent site for nevi and melanoma [11]. A pediatric case of an acquired compound nevus on the palate showed interval enlargement, asymmetry, and color variation before excision, illustrating why change over time matters even when the final diagnosis is benign [12].

Oral melanoacanthoma is an important benign mimic of a clinically suspicious pigmented lesion. It is a reactive melanocytic proliferation that may enlarge rapidly and display dark or irregular pigmentation. A pediatric report described gingival melanoacanthoma in a 17-year-old patient and emphasized its place in the differential diagnosis of gingival hyperpigmentation in young patients [13]. Because its clinical appearance can overlap with melanoma, histopathologic confirmation is appropriate when the diagnosis is uncertain. Recognition of this mimic should support measured counseling of families, not a higher threshold for biopsy.

For a small, stable lesion with a confident benign diagnosis, photographic surveillance may be reasonable. For a new, changing, atypical, or diagnostically uncertain focal melanocytic lesion, referral to oral medicine or oral pathology and histopathologic evaluation should be considered. The choice between excisional and incisional biopsy depends on lesion size, location, and the differential diagnosis; it should not be reduced to a universal "excision first" rule.

Syndromic and endocrine pigmentation

Multifocal or diffuse pigmentation acquires greater significance when accompanied by extraoral findings. A systematic review of syndromic oral pigmented lesions found that oral pigmentation often preceded recognition of the underlying syndrome, supporting the value of a careful oral examination [14]. In children, clinically important considerations include Peutz-Jeghers syndrome (PJS), McCune-Albright syndrome (MAS), primary adrenal insufficiency (Addison disease), and much less commonly, Laugier-Hunziker syndrome (LHS).

PJS should be considered when a child has characteristic mucocutaneous lentigines involving the lips and oral mucosa together with a compatible personal or family history. The clinical importance extends beyond pigmentation because PJS is associated with gastrointestinal hamartomatous polyps and childhood complications such as bleeding and intussusception. Pediatric management guidance emphasizes coordinated gastrointestinal surveillance and syndrome-specific care [15]. Pigmentation is therefore a diagnostic clue, not the treatment target.

Oral pigmentation is also an underrecognized feature of MAS. A case series described oral mucosal pigmentation developing during childhood or early adulthood in patients with characteristic skeletal and endocrine manifestations [16]. In a child with mucosal lentigines plus cafe-au-lait pigmentation, fibrous dysplasia, precocious puberty, thyroid disease, or another hyperfunctioning endocrinopathy, MAS should enter the differential.

LHS is a benign acquired mucocutaneous pigmentation disorder that can mimic PJS and adrenal disease. Pediatric presentations are rare; a pediatric case review emphasized that LHS is a diagnosis of exclusion after more consequential causes of mucocutaneous pigmentation have been considered [17]. Clinicians should therefore avoid labeling a child with LHS solely on visual appearance.

Primary adrenal insufficiency can produce generalized cutaneous and mucosal hyperpigmentation through increased adrenocorticotropic hormone and related proopiomelanocortin-derived peptide signaling at melanocortin receptors. Oral pigmentation may accompany fatigue, weight loss, gastrointestinal symptoms, salt craving, hypotension, or electrolyte abnormalities [18]. Suspicion of adrenal insufficiency warrants prompt medical evaluation because the clinical stakes are substantially higher than the oral finding itself.

Medication-related pigmentation

Medication history is essential in any child or adolescent with newly acquired diffuse or multifocal pigmentation. A systematic review of medication-induced oral hyperpigmentation identified several implicated drug classes, including antimicrobials, antimalarials, antineoplastic agents, and antiretroviral therapies; most of the evidence, however, was derived from adults [19].

The mechanism varies by drug and may involve melanin stimulation, deposition of drug or metabolites, iron deposition, or post-inflammatory change. A useful clinical sequence is to establish the temporal relationship between medication exposure and pigmentation, examine whether the distribution matches reported patterns, assess for other mucocutaneous changes, and discuss suspected adverse drug effects with the prescribing clinician. Medically necessary therapy should not be stopped solely because of pigmentation without appropriate clinical review.

Exogenous pigmentation and dental causes

Blue-gray oral pigmentation can result from exogenous material. In children, graphite implantation after pencil trauma is a classic example described in oral-pigmentation reviews [8]. Amalgam tattoo is more typical after restorative dental procedures and is therefore less common in young children than in adults, but it remains relevant in older children with a compatible dental history. A blue-gray macule adjacent to a restored tooth is suggestive; radiography may demonstrate radiopaque particles, although a negative radiograph does not exclude the diagnosis [3,20].

A convincing history plus a compatible location can support conservative management, whereas an unexplained blue-gray or black lesion without a clear dental or traumatic source should not be assumed to be exogenous. When uncertainty remains, oral medicine or oral pathology assessment and biopsy may be necessary to distinguish foreign material from a melanocytic lesion.

Vascular lesions that mimic pigmentation

Red, purple, and blue lesions may represent vascular tumors or malformations rather than melanin pigmentation. Diascopy can be useful because blanching under pressure supports an intravascular component; however, a systematic review found that diascopy and related noninvasive techniques are adjuncts rather than definitive tests, and a negative blanching response does not exclude a vascular lesion [21]. Other noninvasive tools, including dermoscopy, may provide additional structural information in experienced hands, but pediatric validation remains limited and they should not delay tissue diagnosis when a melanocytic lesion remains uncertain [21].

Pediatric oral hemangiomas are uncommon but clinically important. A scoping review emphasized the roles of history, examination, imaging when indicated, and multidisciplinary management [22]. The timing and pattern of growth are useful in distinguishing a proliferating vascular tumor from a vascular malformation. A compressible or pulsatile lesion, a lesion with a history of enlargement, or one in which vascular anatomy is uncertain should not undergo blind office biopsy because of bleeding risk. Referral to a team experienced in pediatric vascular anomalies is appropriate when the diagnosis or treatment plan is uncertain.

Recognizing lesions that require biopsy

The central safety question is not whether melanoma is common in children - it is exceptionally rare - but whether a lesion can be confidently explained as benign. Adult and mixed-age reviews consistently recommend histopathologic assessment when the clinical diagnosis of a focal pigmented lesion remains uncertain [1-4]. Oral mucosal melanoma is an aggressive malignancy whose clinical appearance can include a macule, patch, plaque, or mass with variable pigmentation [23]. A 2026 systematic review and meta-analysis of 16 studies comprising 1,151 patients reported a pooled five-year survival proportion of 34% and reinforced the importance of timely diagnosis [24].

Features that should lower the threshold for specialist evaluation include recent onset, rapid enlargement, new nodularity, ulceration, spontaneous bleeding, irregular or poorly defined borders, marked asymmetry, color variegation, unexplained focal pigmentation on the palate or gingiva, and associated cervical lymphadenopathy. These features are not individually diagnostic of malignancy, and their absence does not guarantee benignity. Oral melanoacanthoma demonstrates why red flags are escalation triggers rather than a surrogate diagnosis. The purpose of a red-flag list is to prompt timely specialist assessment and histopathology when indicated [1-4,23].

The proposed pediatric diagnostic pathway integrates these principles into a history-first, pattern-based approach (Figure 1).

**Figure 1:**
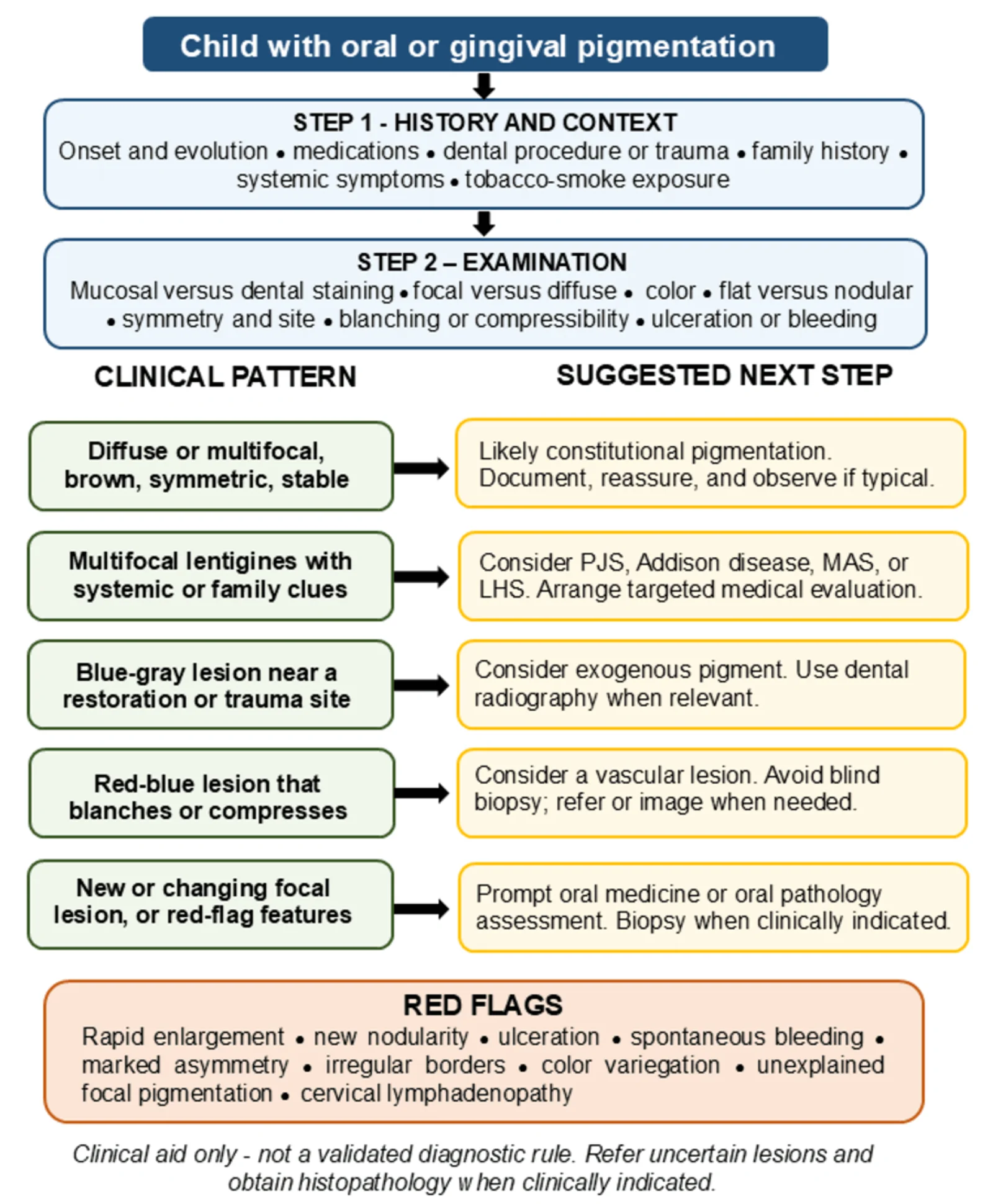
Stepwise diagnostic approach to oral pigmentation in children PJS: Peutz-Jeghers syndrome; MAS: McCune-Albright syndrome; LHS: Laugier-Hunziker syndrome. Flowchart summarizing the proposed clinical sequence and synthesized from the clinical principles described in references [1-4,23]. The diagnostic pathway is intended as a clinical aid and has not been prospectively validated. Image Credit: Created by Ganbat D using Microsoft PowerPoint (Microsoft Corp., Redmond, WA, USA).

It deliberately separates vascular lesions from melanocytic lesions before biopsy and treats syndromic clues as triggers for targeted medical referral rather than as purely oral diagnoses.

Management and follow-up

Management should be proportional to diagnostic confidence and clinical risk. Typical constitutional pigmentation generally requires no treatment. Standardized intraoral photographs can provide a useful baseline when future comparison is likely to influence management. Solitary lesions with a benign but not definitive clinical impression may require specialist review or histopathology, whereas systemic patterns require syndrome-directed evaluation rather than local treatment.

Cosmetic depigmentation should be approached cautiously in children. Pediatric case reports describe surgical depigmentation, and a broader systematic review and network meta-analysis has compared laser and surgical approaches for oral hyperpigmentation [25,26]. Most treatment evidence is not pediatric-specific, recurrence can occur, and physiologic pigmentation is not a disease. In minors, treatment decisions should therefore include a clear discussion of benign natural history, the elective nature of intervention, procedural morbidity, recurrence, and the child's own preferences in addition to parental concerns.

Table 1 summarizes the main differential diagnoses and first-line actions, and Table 2 provides a risk-tiered management framework for pediatric dental and medical practice.

**Table 1:** Clinical differential diagnosis of oral pigmentation in children Principal differential diagnoses and corresponding first-line clinical actions. GI: gastrointestinal.

Condition or category	Typical oral pattern	Associated clues	First-line action
Constitutional melanin pigmentation	Diffuse or multifocal; bilateral and symmetric; brown to dark brown; stable	No systemic symptoms; often correlates with constitutive skin pigmentation	Document; reassure; photograph if follow-up is useful [3,6,8]
Post-inflammatory melanosis	Brown macule or patch at a site of prior inflammation or trauma	Preceding ulcer, trauma, or mucosal disease	Treat the underlying cause; observe if the diagnosis is secure [1,2]
Melanotic macule	Solitary, flat, well-circumscribed brown macule	Usually asymptomatic; may be labial, gingival, or palatal	Observe only if diagnosis is secure; biopsy if new, changing, atypical, or uncertain [10,11]
Melanocytic nevus	Focal brown, blue, or black macule or papule; uncommon in oral mucosa	Palate is a recognized site; clinical overlap with other melanocytic lesions	Oral medicine or oral pathology review; biopsy when clinically indicated [11,12]
Oral melanoacanthoma	Dark macule or patch that may enlarge rapidly	Benign reactive lesion; may appear clinically alarming	Biopsy when uncertain to exclude melanoma; counsel after histopathologic confirmation [13]
Peutz-Jeghers syndrome (PJS)	Multiple perioral and intraoral lentigines	Family history; GI polyps; bleeding or intussusception risk	Genetics and gastroenterology referral with syndrome-specific surveillance [14,15]
McCune-Albright syndrome	Mucosal lentigines, often developing after early childhood	Cafe-au-lait lesions; fibrous dysplasia; hyperfunctioning endocrinopathies	Endocrine and genetic evaluation when the phenotype is compatible [16]
Primary adrenal insufficiency	Diffuse mucosal and cutaneous hyperpigmentation	Fatigue, weight loss, GI symptoms, salt craving, hypotension, electrolyte abnormalities	Prompt medical or endocrine assessment [18]
Laugier-Hunziker syndrome	Acquired multifocal oral and labial pigmentation	Usually no systemic disease; rare in children; diagnosis of exclusion	Exclude PJS, adrenal disease, and other causes before diagnosis [17]
Medication-induced pigmentation	Acquired focal or diffuse pigmentation; pattern varies by drug	Temporal relationship to medication; possible skin or nail changes	Review medications with prescriber; do not stop essential therapy without medical advice [19]
Exogenous pigment	Blue-gray or black focal macule	Restoration, dental procedure, or penetrating trauma history	Radiograph when relevant; biopsy if the source remains uncertain [3,20]
Vascular lesion	Red, blue, or purple; may blanch or compress	Growth history, pulsation, or soft compressibility	Avoid blind biopsy; specialist referral and imaging when needed [21,22]
Indeterminate or suspicious focal pigmented lesion	New or changing focal lesion; asymmetry, variegation, nodularity, ulceration, or bleeding	May otherwise be asymptomatic	Prompt specialist assessment and biopsy when indicated; oncology referral if malignancy is confirmed [23,24]

**Table 2:** Risk-tiered management framework for oral pigmentation in children Risk tiers guide the intensity of investigation and referral and do not replace clinical judgment. A child may move between tiers as the lesion evolves. GI: gastrointestinal.

Risk tier	Clinical pattern	Recommended action
Low risk	Longstanding, symmetric, flat, stable pigmentation with a convincing benign explanation	Reassurance; baseline documentation; routine review
Intermediate risk	New focal lesion without alarming features; uncertain exogenous source; atypical but stable lesion	Oral medicine or oral pathology referral; targeted imaging or biopsy according to specialist assessment
Systemic-pattern risk	Multifocal lentigines or diffuse pigmentation with GI, endocrine, skeletal, family-history, or medication clues	Targeted medical referral and syndrome or drug evaluation; local treatment is secondary
Vascular-pattern risk	Red-blue lesion that blanches or compresses, is pulsatile, or has suspected vascular anatomy	Avoid blind biopsy; vascular-anomaly referral and imaging when indicated
High risk	Rapid growth, nodularity, ulceration, spontaneous bleeding, irregular borders, marked asymmetry, color variegation, unexplained focal lesion, or lymphadenopathy	Expedited specialist assessment and histopathologic diagnosis

Limitations and future research

This review has important limitations. It is a narrative synthesis rather than a systematic review, sources were selected for clinical relevance rather than exhaustive quantitative synthesis, no formal risk-of-bias assessment was performed, and the pediatric evidence base remains limited. Many recommendations are supported by adult oral-medicine literature, small pediatric observational studies, case reports, or case series. The prevalence and natural history of specific pigmented lesions also vary by population, skin phototype, age, and assessment method. Consequently, the diagnostic pathway should not be interpreted as a validated scoring system.

Prospective pediatric studies are needed to define the natural history of constitutional pigmentation and oral nevi, evaluate standardized photographic or colorimetric monitoring, and assess the diagnostic performance and interobserver reliability of proposed red-flag criteria. Multicenter registries would be particularly valuable for rare but high-consequence conditions such as pediatric oral mucosal melanoma. Studies of noninvasive imaging, including dermoscopy, should determine whether these tools can safely reduce unnecessary biopsy without delaying diagnosis of clinically important lesions.

## Conclusions

Oral pigmentation in children is most often benign, but the same visual finding can occasionally indicate a medication effect, exogenous material, a vascular lesion, a genetic syndrome, an endocrine disorder, or a melanocytic neoplasm. A structured evaluation based on onset, evolution, distribution, color, surface characteristics, blanching, dental and medication history, family history, and systemic findings provides a practical way to narrow the differential diagnosis.

The safest clinical strategy is to reassure only when the pattern is convincingly benign, refer when systemic or vascular clues are present, and obtain specialist assessment with histopathologic evaluation when a focal lesion is new, changing, atypical, or cannot be confidently classified. The proposed framework is intended to support consistent pediatric practice while recognizing that clinical judgment and histopathology remain decisive when uncertainty persists.
